# Randomized phase II study of paclitaxel/carboplatin intercalated with gefitinib compared to paclitaxel/carboplatin alone for chemotherapy-naïve non-small cell lung cancer in a clinically selected population excluding patients with non-smoking adenocarcinoma or mutated EGFR

**DOI:** 10.1186/s12885-015-1714-y

**Published:** 2015-10-22

**Authors:** Yoon Ji Choi, Dae Ho Lee, Chang Min Choi, Jung Shin Lee, Seung Jin Lee, Jin-Hee Ahn, Sang-We Kim

**Affiliations:** 1 Department of Oncology, Asan Medical Center, University of Ulsan College of Medicine, 88 Olympic-ro-43-gil, Songpa-gu, Seoul, 138-736 Korea; 2 Division of Hemato-oncology, Department of Internal Medicine, Korea University Anam Hospital, Seoul, Korea; 3 Asan Institute for Life Science, Asan Medical Center, Institute for Innovative Cancer Research, Seoul, Korea; 4 University of Ulsan College of Medicine, Ulsan, Korea; 5 Department of Oncology, Asan Medical Center, University of Ulsan College of Medicine, 88 Olympic-ro 43-gil, Songpa-gu, Seoul, 138-736 Korea

**Keywords:** Non-small cell lung cancer, Intercalated chemotherapy, Gefitinib, Smoker, Wild-Type EGFR

## Abstract

**Background:**

Considering cell cycle dependent cytotoxicity, intercalation of chemotherapy and epidermal growth factor receptor (EGFR) tyrosine kinase inhibitor (TKI) may be a treatment option in non-small cell lung cancer (NSCLC). This randomized phase 2 study compared the efficacy of paclitaxel and carboplatin (PC) intercalated with gefitinib (G) versus PC alone in a selected, chemotherapy-naïve population of advanced NSCLC patients with a history of smoking or wild-type EGFR.

**Methods:**

Eligible patients were chemotherapy-naïve advanced NSCLC patients with Eastern Cooperative Oncology Group performance status of 0—2. Non-smoking patients with adenocarcinoma or patients with activating EGFR mutation were excluded because they could benefit from gefitinib alone. Eligible patients were randomized to one of the following treatment arms: PCG, P 175 mg/m^2^, and C AUC 5 administered intravenously on day 1 intercalated with G 250 mg orally on days 2 through 15 every 3 weeks for four cycles followed by G 250 mg orally until progressive disease; or PC, same dosing schedule for four cycles only. The primary endpoint was the objective response rate (ORR), and the secondary endpoints included progression-free survival (PFS), overall survival (OS), and toxicity profile.

**Results:**

A total of 90 patients participated in the study. The ORRs were 41.9 % (95 % confidence interval (CI) 27.0–57.9 %) for the PCG arm and 39.5 % (95 % CI 25.0–55.6 %) for the PC arm (*P* = 0.826). No differences in PFS (4.1 vs. 4.1 months, *P* = 0.781) or OS (9.3 vs. 10.5 months, *P* = 0.827) were observed between the PCG and PC arms. Safety analyses showed a similar incidence of drug-related grade 3/4 toxicity. Rash and pruritus were more frequent in the PCG than in the PC arm.

**Conclusions:**

PCG did not improve ORR, PFS, and OS compared to PC chemotherapy alone for NSCLC in a clinically selected population excluding non-smoking adenocarcinoma or mutated EGFR.

**Trial registration:**

The study is registered with ClinicalTrials.gov (NCT01196234). Registration date is 08/09/2010.

## Background

Despite significant advances in the treatment of non-small-cell lung cancer (NSCLC) over the last two decades, the results of standard chemotherapy for advanced NSCLC have reached a plateau and new treatment strategies are necessary. The introduction of epidermal growth factor receptor (EGFR) inhibitors was considered a promising strategy. EGFR-tyrosine-kinase inhibitors (TKIs), specifically gefitinib and erlotinib, are currently considered the standard first-line treatment for patients with activating EGFR mutations based on the results of several randomized studies [1–6]. However, the benefit of these agents is confined to patients with EGFR mutation [7, 8].

The mechanism of action of EGFR inhibitors is the inhibition of tumor cell proliferation and induction of apoptosis. The addition of EGFR inhibitors to standard chemotherapy is an attractive approach to enhance its efficacy. However, no survival advantage was detected in trials such as the INTACT I, II, and TRIBUTE studies [9–11]. One possible explanation for the failure of these studies is that tumor cells that were driven to G0/G1 phase by EGFR TKIs may not be sensitive to cytotoxic chemotherapy. Preclinical studies and several phase 1 and 2 studies showed that sequential treatment with chemotherapy followed by EGFR TKIs led to synergistic cytotoxicity [12–16].

The present study examined the effect of gefitinib administered for 2 weeks after paclitaxel and carboplatin (PC) chemotherapy by assessing cell-cycle progression during chemotherapy in patients with NSCLC. Non-smoking patients with adenocarcinoma and patients with mutant EGFR, who were expected to benefit from gefitinib alone, were excluded from the analysis.

## Methods

### Study design and population

This study was a single-center, prospective, open-label, randomized phase II study of paclitaxel and carboplatin with intercalated gefitinib (PCG) or PC alone (PC) for advanced NSCLC in a selected population of smokers with wild-type EGFR.

Patients were eligible for this study if they were 18 years or older, had a histological diagnosis of NSCLC with metastasis (stage IV) or locally advanced (stage IIIB) disease with malignant pleural effusion according to the 6th edition of the American Joint Committee on Cancer staging system. Inclusion criteria were ≥ 1 measurable lesion meeting Response Evaluation Criteria in Solid Tumors (RECIST version 1.1) guidelines, an Eastern Cooperative Oncology Group (ECOG) performance status (PS) of 0–2, at least 1 week since the last radiotherapy session, and adequate organ function.

Exclusion criteria included tumors harboring EGFR mutation, prior systemic chemotherapy for NSCLC, non-smoking patients with adenocarcinoma (except patients with wild-type EGFR), symptomatic brain metastasis and any unstable medical condition.

This study was approved by the institutional review board of Asan Medical Center and was performed in accordance with the Declaration of Helsinki and Good Clinical Practice guidelines. All patients gave written informed consent before treatment. This study has been submitted for registration with ClinicalTrials.gov identifier NCT01196234.

### Randomization

Eligible patients were randomly assigned in a 1:1 ratio to receive PCG or PC alone. Randomization was stratified by gender and histology (adenocarcinoma, others).

### Treatment plan

Patients in the PCG arm received paclitaxel 175 mg/m^2^ and carboplatin AUC 5 intravenously on day 1 with intercalated gefitinib 250 mg orally once daily from day 2 through day 15 every 3 weeks for four cycles followed by gefitinib 250 mg orally until progressive disease or unacceptable toxicity. Patients in the PC arm received paclitaxel 175 mg/m^2^ and carboplatin AUC 5 intravenously on day 1 every 3 weeks until progressive disease up to four cycles without following maintenance therapy.

Dose adjustments were based on drug-related toxicities. When dose reduction was necessary, gefitinib was stopped every third day. If a patient still required dose reduction, it was stopped every other day. Any patient who required three dose reductions or developed interstitial lung disease (ILD) was discontinued from the study drug. Treatment could be delayed for a maximum of 2 weeks.

### Evaluation

Physical examination, ECOG PS evaluation and toxicity rating according to the Common Terminology Criteria for Adverse Events (CTCAE version 3.0) were performed at baseline, prior to each cycle, and approximately 1 month after the last dose of the study drug. Tumor response was assessed by computed tomography (CT) with RECIST version 1.1 every 6 weeks during chemotherapy, and every 12 weeks thereafter until disease progression.

### Statistical considerations

The aim of this randomized phase II study was to assess the benefit of gefitinib intercalation with PC chemotherapy. The primary endpoint was objective response rate (ORR); complete response, (CR) or partial response, (PR). When the usual ORR in the PC chemotherapy arm was 40 % and an absolute increase of 15 % in the ORR was obtained by intercalation of gefitinib to standard PC chemotherapy in each arm of 37 patients, the probability of correctly selecting PC chemotherapy with gefitinib as superior was 0.9. Considering follow-up loss, 42 patients were planned to be enrolled in each arm [17].

ORR was analyzed using the *χ*2 test and data were expressed with 95 % confidence interval (CI). The secondary endpoints were progression-free survival (PFS) and overall survival (OS), which were assessed using the Kaplan-Meier method with hazard ratio (HR) and 95 % CI via Cox’s proportional hazard model.

## Results

**Fig. 1:**
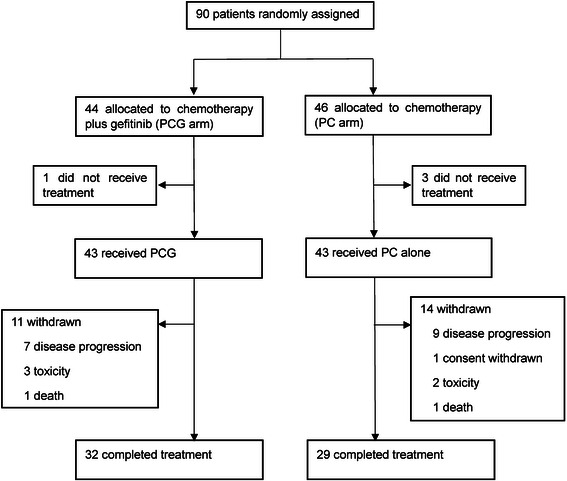
Trial profile

Between April 2010 and December 2011, 90 patients were enrolled into the study and randomly assigned to receive PC with gefitinib (*N* = 44) or PC alone (*N* = 46). Finally, 43 patients in the PCG arm and 43 patients in the PC arm received at least one cycle of treatment (Fig. 1). Baseline characteristics were well balanced across the treatment arms (Table 1). Median age was 60 (range, 44–72) years in the PCG arm and 59 (range, 37–70) years in the PC arm. Approximately 10 % of patients were non-smokers and 63 % had adenocarcinoma. Patients with stage IV cancer comprised about 80 % of each group equally.

**Table 1:** Baseline characteristics of patients (intention to treat population)

Parameter	Chemotherapy-gefitinib (PCG) (*N* = 44) (%)	Chemotherapy (PC) (*N* = 46) (%)	Total (*N* = 90) (%)	*P*-value
Age (years)				
Median (range)	60.0 (44–72)	59.0 (37–70)	59.5 (37–72)	0.678
Gender				
Male	35 (79.5)	42 (91.3)	77 (85.6)	
Female	9 (20.5)	4 (8.7)	13 (14.4)	0.113
Smoking status				
Smoker	37 (84.1)	44 (95.7)	81 (90.0)	
Non-smoker	7 (15.9)	2 (4.3)	9 (10.0)	0.087
ECOG status				
ECOG 0	0	0	0	
ECOG 1	44 (100)	46 (100)	90 (100)	–
Histological subtype				
Adenocarcinoma	24 (54.5)	31 (67.4)	55 (61.1)	
Non-adenocarcinoma	20 (45.5)	15 (32.6)	35 (38.9)	0.211
EGFR mutation				
Wild-type	6 (13.6)	3 (6.5)	9 (10.0)	
Unknown	38 (86.4)	43 (93.5)	81 (90.0)	0.157
Stage of disease				
Stage IIIB	8 (18.2)	10 (21.7)	18 (20.0)	
Stage IV	36 (81.8)	36 (78.3)	72 (80.0)	0.673

### Primary efficacy measures

No significant difference in ORR was observed between the two arms (*P* = 0.826), with an ORR of 41.9 % (95 % CI: 27.0–57.9 %) for the PCG arm and 39.5 % (95 % CI: 25.0–55.6 %) for the PC arm (Table 2). No difference in disease control rate (DCR) was observed between the two arms, with DCR values of 74.4 % (95 % CI: 58.8–86.5 %) and 65.1 % (95 % CI: 49.1–79.0 %), respectively (*P* = 0.348) (Table 2).

**Table 2:** Best overall response according to RECIST

Parameter	PCG arm (%) (*N* = 43)	PC arm (%) (*N* = 43)	Odds ratio (95 % CI)	*P*-value
Objective response	18 (41.9)	17 (39.5)	0.91	0.826
95 % CI	(27.0–57.9)	(25.0–55.6)	(0.38–2.13)	
Disease control	32 (74.4)	28 (65.1)	0.64	0.348
95 % CI	(58.8–86.5)	(49.1–79.0)	(0.25–1.61)	
Complete response	0 (0)	0 (0)		
Partial response	18 (41.9)	17 (39.5)		
Stable disease	14 (32.6)	11 (25.6)		
Progressive disease	10 (23.3)	13 (30.2)		
Missing	1 (2.3)	2 (4.7)		

*N* number; *CI* confidence interval

### Secondary efficacy measures

**Fig. 2:**
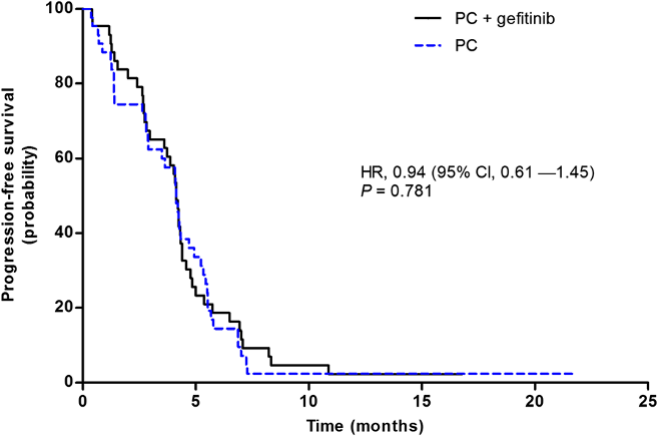
Kaplan-Meier graph of progression-free survival by treatment group (ITT population)

During a median follow-up of 21.7 months, there were 83 patients with PFS events (disease progression or death from any cause). No statistically significant difference in PFS was found between the two arms (HR = 0.94 [95 % CI: 0.61–1.45], *P* = 0.781). Median PFS was 4.1 (95 % CI: 3.9–4.3) months in the PCG arm and 4.1 (95 % CI: 3.9–4.3) months in the PC arm (Fig. 2).

**Fig. 3:**
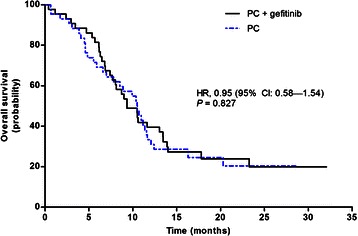
Kaplan-Meier graph of overall survival by treatment group (ITT population)

A total of 66 patients had an OS event (death). No statistically significant difference in OS was observed between the groups (HR = 0.95 [95 % CI: 0.58–1.54], *P* = 0.827). Median OS was 9.3 (95 % CI: 7.0–11.6) months in the PCG arm and 10.5 (95 % CI: 8.3–12.7) months in the PC arm (Fig. 3).

### Exploratory analyses

**Fig. 4:**
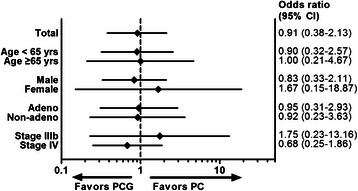
Forest plots by clinical subgroups. yrs, years; adeno, adenocarcinoma

Exploratory subgroup analyses are shown in Fig. 4. The negative result for the comparison between the PCG arm and the PC arm was generally consistent throughout all clinical subsets, although the small number of patients in several subsets resulted in large CIs and made the results difficult to interpret.

### Safety

Of the 90 patients, 86 received at least one dose of treatment and were included in the safety analyses. The median number of cycles received in both arms was 4.0.

The two arms showed a similar incidence of drug-related toxicity. Most AEs were clinically manageable. The most commonly reported AEs of any grade were anemia, neutropenia, rash, pruritus, myalgia, neuropathy, anorexia, and cough (Table 3). Skin rash and pruritus were more common in the PCG arm (63 and 37 %) than in the PC arm (5 and 9 %), although no grade 3 rash or pruritus was observed in the PCG arm (Table 3). Frequency of diarrhea was similar in both treatment groups (Table 3). Twenty-two patients had at least one serious adverse event with CTCAE grade 3/4 [10 in the PCG arm (23.3 %) and 12 in the PC arm (27.9 %)] (Table 3). Three patients died during the study period, two in the PCG arm (both from infection) and one in the PC arm (from pulmonary thromboembolism).

**Table 3:** Summary of the most common adverse events

	PCG arm (*N* = 43) (%)				PC arm (*N* = 43) (%)				*P*-value for all grade AE
	All grade	Gr 3	Gr 4	Gr 5	All grade	Gr 3	Gr 4	Gr 5	
Patients with ≥ 1 AE (Gr3/4/5)	10 (23)				12 (28)				0.244
^a^Rash	27 (63)				2 (5)				<0.001
^a^Pruritis	13 (37)				4 (9)				0.012
Myalgia	25 (58)				28 (65)				0.506
Neuropathy	21 (49)				25 (58)				0.387
Alopecia	24 (56)				21 (49)				0.517
Anorexia	15 (35)				18 (42)				0.506
Cough	16 (37)				11 (26)				0.245
Nausea	13 (30)				8 (19)				0.209
Fatigue	10 (23)				10 (23)				1.000
Dyspepsia	6 (14)				7 (16)				0.763
Constipation	6 (14)				6 (14)				1.000
Diarrhea	5 (12)				5 (12)	1 (2)			1.000
Chest pain	5 (12)				5 (12)				1.000
General weakness	5 (12)	1 (2)			5 (12)				1.000
Infection	6 (14)	4 (9)		2 (5)	3 (7)	3 (7)			0.483
TE event	1 (2)				2 (5)		1 (2)	1 (2)	0.571
Neutropenia	10 (23)		2 (5)		7 (16)	3 (7)	1 (2)		0.660
Febrile neutropenia	0				1 (2)				1.000
Anemia	35 (81)	1 (2)			34 (79)	1 (2)			0.787
Thrombocytopenia	9 (21)				8 (19)	1 (2)			0.787
Leucopenia	6 (14)	2 (5)			6 (14)		1 (2)		1.000
Increased LFT	15 (35)	1 (2)			11 (26)				0.348

*AE* adverse event; *Gr* grade; *N* number, *LFT* liver function test

^a^significant difference between two groups

## Discussion

This randomized phase II study was designed to evaluate the effect of intercalation therapy with gefitinib and paclitaxel/carboplatin chemotherapy as first-line treatment in a clinically selected population, excluding non-smoking patients with adenocarcinoma or patients with wild-type EGFR. Our study demonstrated that gefitinib intercalation did not improve the efficacy of paclitaxel/carboplatin chemotherapy in relation to ORR, PFS, and OS. Toxicity profiles were generally clinically tolerable. Combination treatment resulted in more frequent skin toxicity.

Earlier studies that assessed the combination of chemotherapy and EGFR TKIs failed to show a survival advantage. In two randomized studies, the addition of daily gefitinib or erlotinib to standard chemotherapy did not improve OS, time to progression or ORR compared with chemotherapy alone [9–11].

Two possible combination approaches have been proposed to solve this problem: a pure sequential strategy, in which chemotherapy is followed by maintenance EGFR TKI treatment [18, 19], and an intercalated administration strategy based on cell cycle-dependent cytotoxicity, which was supported by the results of preclinical and preliminary clinical studies [12–16].

One preclinical study assessed the effects of sequential administration of pemetrexed and erlotinib, and showed cytotoxic synergism in both mutant and wild-type EGFR cell lines [12]. In another preclinical study, the sequence-dependent synergism between paclitaxel and gefitinib was demonstrated in human lung cancer cell lines with both wild-type and mutant EGFR genes [13]. Several later phase I/II clinical studies showed that an intercalated regimen of chemotherapy and EGFR TKI is safe and effective [14–16, 20].

Recently, two clinical studies reported that the intercalated regimen offered superior efficacy compared to chemotherapy or EGFR TKIs alone [21, 22]. In the First-line Asian Sequential Tarceva and Chemotherapy Trial (FASTACT)-2, intercalated therapy with gemcitabine plus platinum and erlotinib improved OS and PFS compared to chemotherapy alone for unselected patients with advanced stage NSCLC as first-line setting. In subset analyses, patients with wild-type EGFR did not benefit from this intercalated regimen. [21] In a three-arm phase II study, pemetrexed-erlotinib improved PFS compared to either drug alone in a clinically selected population of never-smoking patients with non-squamous NSCLC as second-line therapy [22].

Because the combination of chemotherapy and EGFR TKIs showed cytotoxic synergism against wild-type EGFR NSCLC cell lines in a preclinical study [12, 13] and this combination was suggested as a new treatment option for patients with unknown EGFR status in a previous clinical study [21], we hypothesized that the intercalated strategy could be effective in patients with wild-type or unknown EGFR status. Despite the results of preclinical and clinical studies, our study failed to show the efficacy of intercalated therapy in patients with wild-type EGFR or in a clinically selected population that excluded non-smoking patients with adenocarcinoma. Although molecular tests are used routinely in clinical practice, EGFR status remains unknown in certain patients. The negative result of the present study was consistent with the results of Matjaz Zwitter et al.’s study, which showed that intercalated treatment was not of benefit for EGFR wild-type NSCLC [23].

On the other hand, intercalated treatment might be a promising approach for patients with NSCLC with EGFR mutant disease or selected patient with unknown EGFR mutation status, according to several clinical studies [21–23]. There were some explanations for the high efficacy of the intercalated therapy, including synergism of different categories of drugs and preventing repopulation of the tumor. However, a randomized trial comparing intercalated therapy with sequential treatment is needed to confirm the real value of intercalated therapy for EGFR mutated NSCLC.

## Conclusions

In conclusion, the results of the present study indicated that intercalated treatment with chemotherapy and EGFR TKIs does not improve ORR, PFS, and OS compared to chemotherapy alone in patients in a clinically selected population excluding patients with non-smoking adenocarcinoma or mutated EGFR.

## Abbreviations

EGFR: Epidermal growth factor receptor
TKI: Tyrosine kinase inhibitor
NSCLC: Non-small cell lung cancer
PCG: Paclitaxel/carboplatin with gefitinib
PC: Paclitaxel/carboplatin
ORR: Objective response rate
PFS: Progression-free survival
OS: Overall survival
CI: Confidence interval
ECOG: Eastern Cooperative Oncology Group
PS: Performance status
AE: Adverse event
ITT: Intention-to-treat
